# The first case report of CODAS syndrome in Chinese population caused by two *LONP1* pathogenic mutations

**DOI:** 10.3389/fgene.2022.1031856

**Published:** 2023-01-04

**Authors:** Yi Tang, Yu-Xing Liu, Yue Sheng, Liang-Liang Fan, Ai-Qian Zhang, Zhao-Fen Zheng

**Affiliations:** ^1^ Department of Cardiology, Hunan Provincial People’s Hospital, The First Afliated Hospital of Hunan Normal University, Clinical Medicine Research Center of Heart Failure of Hunan Province, Hunan Normal University, Changsha, China; ^2^ Department of Cell Biology, School of Life Sciences, Central South University, Changsha, China; ^3^ Department of Obstetrics and Gynecology, Third Xiangya Hospital of Central South University, Changsha, China

**Keywords:** CODAS syndrome, LONP1, mutation, whole-exome sequencing, compound heterozygote

## Abstract

**Background:** CODAS syndrome (MIM 600373) is a multi-system developmental disorder characterized by cerebral, ocular, dental, auricular, and skeletal anomalies. CODAS syndrome is rare in the world and no cases have been reported in Chinese population so far. Mutations in the *LONP1* gene can contribute to CODAS syndrome, while the underlying molecular mechanisms requires further investigation.

**Method:** We described a Chinese boy who has suffered from cognition impairment, cataracts, caries, abnormal auricle and skeletal anomalies since birth. The patient’s parents are non-consanguineous and healthy. Whole-exome sequencing (WES) was employed to explore the genetic entity of this family.

**Results:** A compound heterozygous missense mutation (NM_004793: c.2009C>T/p.A670V and c.2014C>T/p.R672C) of *LONP1* was identified in the patient. Considering the clinical phenotypes and genetic results, the patient was diagnosed as CODAS syndrome.

**Conclusion:** Here we reported the first case with CODAS syndrome in Chinese population. WES identified a compound heterozygous missense mutation of *LONP1* gene in the patients. Our study not only provided data for genetic counseling and clinical diagnosis to this family, but also expanded the clinical spectrum of *LONP1*-related CODAS syndrome.

## Introduction

CODAS syndrome (MIM 600373), described by Shebib et al. (1991), in 1991, is a multi-system developmental disorder characterized by cerebral, ocular, dental, auricular, and skeletal anomalies. Since it is a rare disease, over the past three decades, only a few cases were reported as CODAS syndrome all around the world (Khan and AlBakri, 2018; Patel et al., 2017; Inui et al., 2017; Dikoglu et al., 2015; Yoo et al., 2019). Most of the cases have been reported from Europe, the Americas and Saudi Arabia. The existing cases in Asia have been reported from Japan and South Korea (Dikoglu et al., 2015). So far, no case has been reported as CODAS syndrome in Chinese population.

The phenotypes of CODAS syndrome that have been reported so far including hypotonia and motor delay, intellectual disability, epilepsy, growth retardation, craniofacial deformities, cataracts, ptosis, delayed tooth eruption, enamel dysplasia, dens hypoplasia, scapha and helix dysplasia, scapha and helix dysplasia, conductive or sensorineural hearing loss, short stature, skeletal dysplasia, scoliosis, genu valgus, pes valgus, vertebral coronal clefts and so on (Strauss et al., 2015). The clinical spectrum of CODAS syndrome may be larger than the typical form described in existing case reports and might be further expanded as more new cases were reported (Dikoglu et al., 2015; Yoo et al., 2019).

CODAS syndrome is an autosomal recessive inherited disease. Bi-allelic mutations in the nuclear gene *LONP1* (homozygous or compound heterozygous) can cause CODAS syndrome. *LONP1* encodes a mitochondrial protease that acts as a key modulator of protein quality control, respiratory complex assembly, gene expression and stress responses in mitochondria (Strauss et al., 2015; Venkatesh et al., 2012). Genetic defects in the *LONP1* gene may cause various mitochondrial disorders and interfere with homeostasis. Patients with bi-allelic mutations of *LONP1* have various clinical defects, some of which are common to mitochondrial disorders. In addition to classic mitochondrial disease presentation, some patients also have marked skeletal abnormalities, which are pathognomonic for CODAS syndrome (Peter et al., 2018). Nevertheless, the exact molecular mechanism how *LONP1* mutations leading to CODAS syndrome is not fully understood. Also, it is still not clear why phenotypes of CODAS syndrome caused by *LONP1* mutations are diverse.

In the present study, we reported a Chinese boy who has suffered from cognition impairment, cataracts, caries, abnormal auricle and skeletal anomalies since birth. The patient’s parents are non-consanguineous and healthy. Whole exome sequencing (WES) and Sanger sequencing were applied to explore the candidate genes of this family.

## Case presentation

**FIGURE 1:**
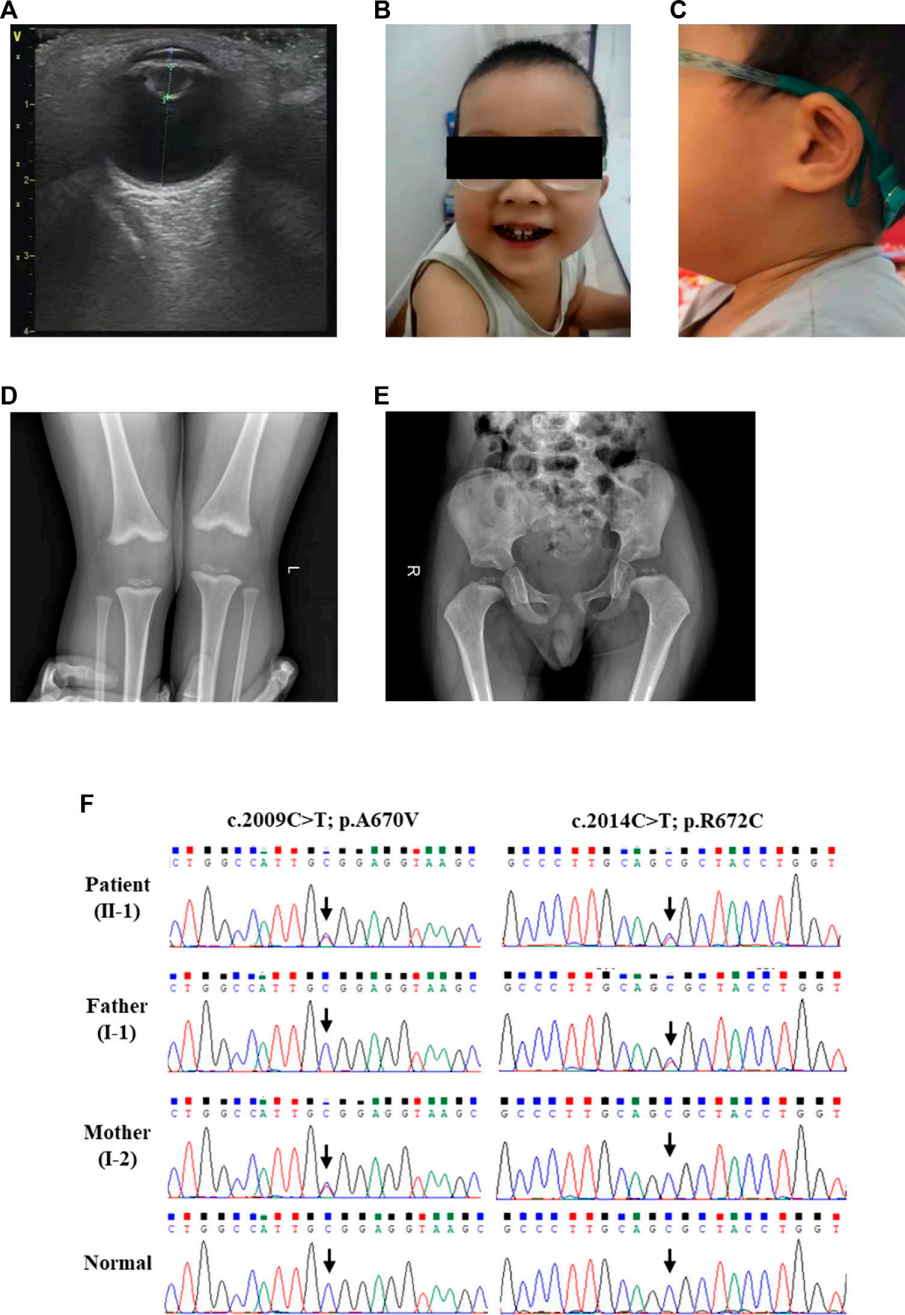
The clinical and sequencing data of this patient. **(A)** Ultrasonic examination of the patient’s eye. Physical features of this patient included **(B)** caries and **(C)** the auricles of both ears were small, and the contour was abnormally folded. **(D)** Skeletal radiography of the patient’s knees. **(E)** The pelvic radiographs of the patient. **(F)** Sanger DNA sequencing chromatogram detected a compound heterozygous missense mutation (NM_004793: c.2009C>T/p.A670V and c.2014C>T/p.R672C) of *LONP1* gene in the patient.

This three-year-old boy was born at term (38 + 5 weeks) with a birth weight of 3.75 kg to healthy Han-Chinese parents (29 years) of non-consanguineous marriage. Neither parent has a family history of genetic disease. At the age of 2 months, his parents found he did not make eye contact. Subsequently, the boy was diagnosed with cataracts (Figure 1A), nystagmus and undergo binocular lens extraction. His teeth began to grow at 9 months, and caries developed at 2 years old (Figure 1B). The auricles of both ears were small, and the contour was abnormally folded (Figure 1C), but the hearing was normal. He also had mild speech delay and dysarthria. His intelligence and athletic ability were clearly behind his peers. He had problems with cognitive functioning, such as attention and learning. But the Magnetic resonance imaging (MRI) at aged 2 years did not find any impairment. He started to stand without support at 15 months and walked without assistance at 18 months. His balance function and posture stability were also not good. The X-ray at 2 years suggested knee valgus (Figure 1D), small, irregular and cracked femoral epiphyseal ossification center (Figure 1E). Routine hematologic and biochemical tests were within the normal range except the high levels of total cholesterol (7.97 mmol/L) and low-density lipoprotein (4.94 mmol/L). At present, he started to receive comprehensive rehabilitation–physical, occupational, and speech-language therapies.

## Genetic analysis

**FIGURE 2:**
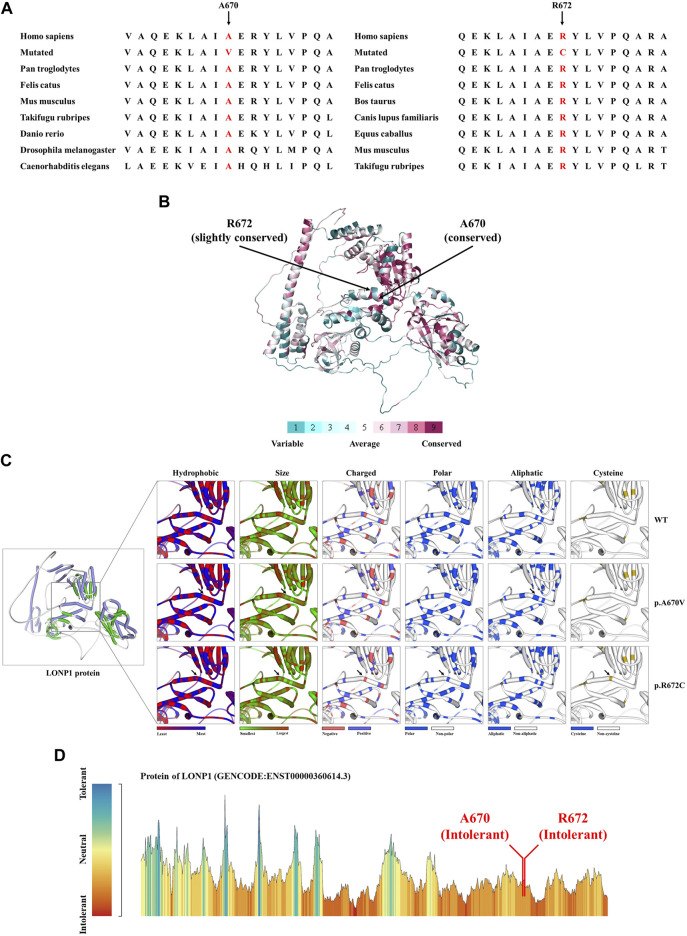
The bioinformatics analysis of mutations. **(A)** Alignment of multiple LONP1 protein sequences across species. Letters in red show both the A670 site and the R672 are evolutionarily conserved. **(B)** The conservation analysis of the A670 site and the R672 amino acids were predicted by ConSurf Server software. **(C)** Structure prediction of wild type protein and mutant proteins. The wild type LONP1 (LONP1-WT) protein structure, the p.A670V mutant LONP1 (LONP1-p.A670V) protein structure and the p.R672C mutant LONP1 (LONP1-p.R672C) protein structure were predicted by SWISS-MODEL online software. Black arrows show the changes of LONP1 protein. **(D)** MetaDome server was used to identify the intolerant regions in DES. As depicted, the affected nucleotides/residues are located in an intolerant region.

WES was applied to explore the pathogenic factor of this patient. After data filtration, Sanger sequencing validation and co-separation analysis, a compound heterozygous missense mutation (NM_004793: c.2009C>T/p.A670V and c.2014C>T/p.R672C) of LONP1 was identified in the patient (Figure 1F). According to ACMG guideline (Richards et al., 2015), both mutations belong to pathogenic [PS1 (same amino acid change as a previously established pathogenic variant regardless of nucleotide change)+PS4 (the prevalence of the variant in affected individuals is significantly increased compared with the prevalence in controls)+PM1 (located in a mutational hot spot and critical and well-established functional domain without benign variation)+PM2 (absent from controls in Exome Sequencing Project,1000 Genomes Project or Exome Aggregation Consortium)+PM3 (for recessive disorders, detected in trans with a pathogenic variant)+PP1 (cosegregation with disease in multiple affected family members in a gene definitively known to cause the disease)+PP3 (multiple lines of computational evidence support a deleterious effect on the gene or gene product)+PP4 (patient’s phenotype or family history is highly specific for a disease with a single genetic etiology)]. Although both mutations were reported in previous case reports, the pathogenicity analysis was absent (Dikoglu et al., 2015). We further performed bioinformatics analysis of the pathogenicity of the two mutations. Alignment of LONP1 amino acid sequences showed that both the affected amino acids A670 and R672 are conserved among different species (Figure 2A). At the same time, ConSurf Server software (http://consurf.tau.ac.il/) predicted that two affected amino acids were conserved (Figure 2B). Moreover, SWISS-MODEL software (https://swissmodel.expasy.org/) was utilized to explore the spatial configuration of two mutations (Figure 2C). Furthermore, MetaDome software (https://stuart.radboudumc.nl/metadome/dashboard) indicated that two affected residues are located in the intolerant region of LONP1 (Figure 2D). Considering the clinical phenotypes and genetic results, the patient was diagnosed as CODAS syndrome.

## Discussion

Here we reported a Chinese boy who has suffered from cognition impairment, cataracts, caries, abnormal auricle and skeletal anomalies since birth. The patient’s parents are non-consanguineous and healthy. WES was conducted to evaluate the genetic cause of this family. A compound heterozygous missense mutation (NM_004793: c.2009C>T/p.A670V and c.2014C>T/p.R672C) of *LONP1* was identified in the patient. Sanger sequencing subsequently confirmed that compound heterozygous mutations were parental segregation. The p.A670V heterozygous mutation was maternal and the p.R672C heterozygous mutation was paternal. Thus, the patient was further diagnosed as CODAS syndrome. Our study further confirms that compound heterozygous mutation of *LONP1* is associated with CODAS syndrome.

**FIGURE 3:**
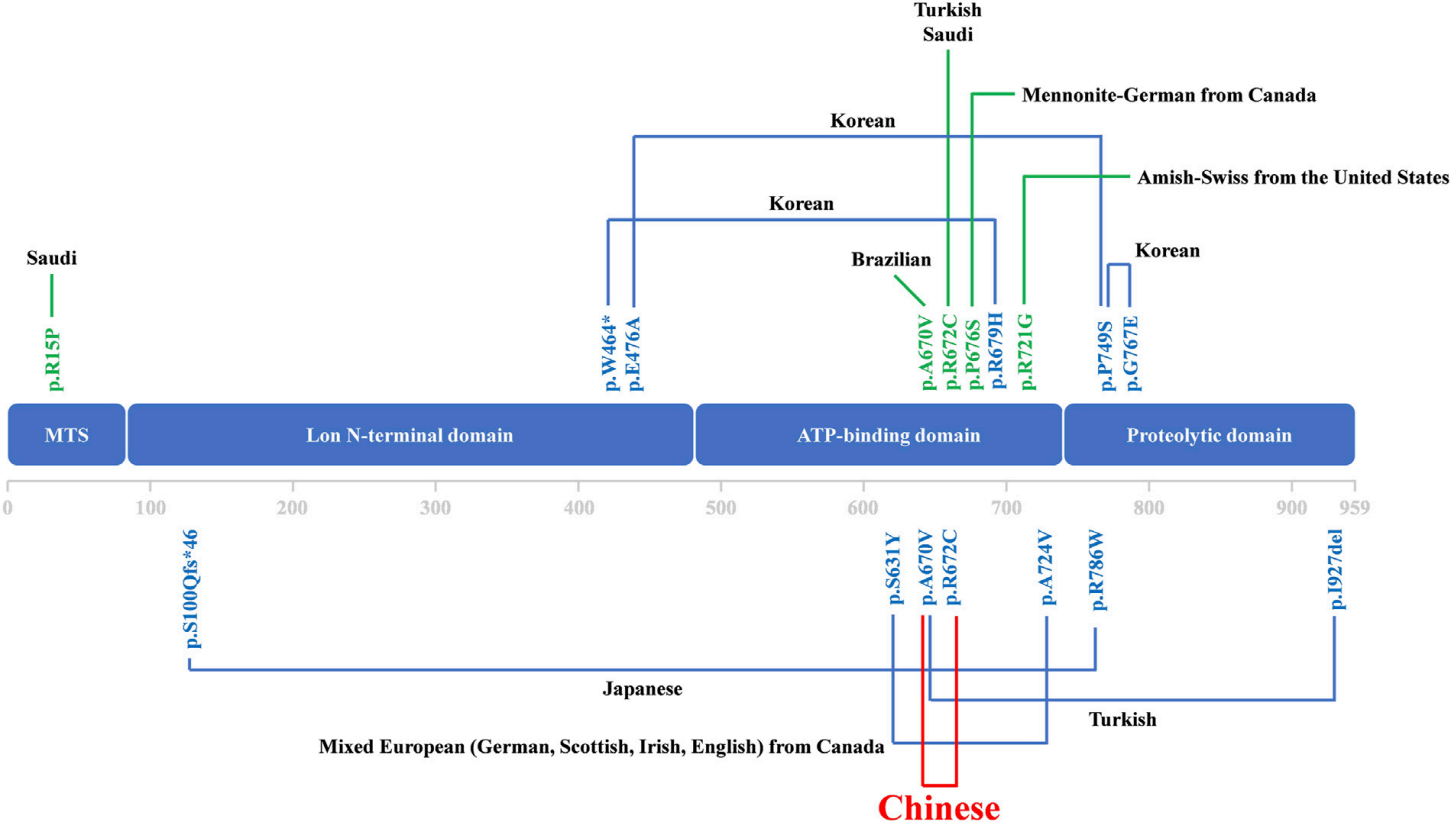
Overview of all reported CODAS syndrome cases caused by *LONP1* mutations.

Over the past three decades, only a few cases were reported as CODAS syndrome all around the world (Khan and AlBakri, 2018; Patel et al., 2017; Inui et al., 2017; Dikoglu et al., 2015; Yoo et al., 2019). Most of the cases have been reported from Europe, the Americas and Saudi Arabia. Some CODAS syndrome individuals from different ancestral backgrounds, such as Amish-Swiss from the United States, Mennonite-German from Canada and mixed European from Canada, have also been reported (Strauss et al., 2015). The existing cases in Asia have been reported from Japan and South Korea (Dikoglu et al., 2015; Inui et al., 2017; Khan and AlBakri, 2018; Patel et al., 2017). According to the reports so far, we may report the first Chinese case who was diagnosed as CODAS syndrome (Figure 3). The phenotype of CODAS syndrome is clinically heterogeneous and the severity of symptoms has a wide range, which brings challenges to clinical diagnosis. Some cases displayed a milder skeletal-ocular only form and did not share enough of the distinctive features to make a diagnosis. Some cases displayed a form of CODAS plus other malformations and might interfere with the diagnosis (Dikoglu et al., 2015; Marlin et al., 2010). CODAS syndrome is rare clinically for these reasons. Because of the sample of the reported CODAS patients is too small, it is hard to explore the frequency of each phenotype or determine which feature should be considered classic for the CODAS syndrome. At present, no clear genotype-phenotype correlation has yet been proposed. Here, we identified a compound heterozygous mutation (NM_004793: c.2009C>T/p.A670V and c.2014C>T/p.R672C) of *LONP1* in a patient with CODAS syndrome. We also supplied the typical clinical pictures of this disease, which may contribute to the understanding and diagnosis of the disease combined with genetic analysis. Similarly, different combinations of p.A670V and p.R672C variants have been already reported in CODAS patients by Dikoglu et al. (2015) in their study, two patients were homozygotes for p.A670V and p.R672C, respectively. And one patient was compound heterozygotes (p.A670V and p.I927del). In our study, the patient was compound heterozygotes (p.A670V and p.R672C). And this combination of variants has not been reported before. We speculated that different combinations of compound heterozygous mutations resulted in phenotypic differences in different cases. Furthermore, a relatively comprehensive review of previously reported CODAS syndrome cases with most characterized *LONP1* variants is showed in Table 1 and may help to establish a genotype-phenotype correlation. In 2015, Strauss et al. (2015) identified that homozygous mutation and compound heterozygous mutation in LONP1 was the genetic lesion of CODAS syndrome. In their study, functional analysis found that pathogenic LONP1 protein showed substrate-specific defects in ATP-dependent proteolysis. The LONP1 variant (c.2161C>G; p.Arg721Gly) homo-oligomerizes poorly *in vitro*. Lymphoblastoid cells generated from patients showed swollen mitochondria with electron-dense inclusions, abnormal inner-membrane morphology, aggregated mtDNA-encoded subunit II of cytochrome C oxidase and reduced spare respiratory capacity, leading to impaired mitochondrial proteostasis and function. The LONP1 encodes a mitochondrial protein which is a member of the Lon family of adenosine triphosphate (ATP)-dependent proteases. The LONP1 holoenzyme forms a ring-shaped homo-hexamer with each subunit mainly consists of a Lon N-terminal domain, an ATP-binding domain, and a proteolytic domain (Qiao et al., 2021; Venkatesh et al., 2012). Previous studies have indicated that this protein can mediate the protein quality control, respiratory complex assembly, gene expression and stress responses in mitochondria (Smakowska et al., 2014; Strauss et al., 2015; Wohlever et al., 2014). Hence, mutations of *LONP1* gene may disrupt the structure and function of mitochondria, which finally induce multi-system developmental disorder. Multiple studies have confirmed that dysfunction of LONP1 activity is one of the important molecular basis of CODAS syndrome. And these mutations usually cluster at the ATP-binding and proteolytic domains of the LONP1 enzyme (Qiao et al., 2021). In agreement with those of previous researches, both the p.A670V mutation and p.R672C mutation reported in this study are located in the ATP-binding domain of LONP1 protein. As shown in the Figure 2C, mutations may cause various changes, including hydrophobic, size, charged, polar, aliphatic and cysteine index, in two affected residues of LONP1 protein, respectively. And these may affect its function as a mitochondrial proteolytic enzyme. While the details of pathogenesis remain to be further explored. Additional functional analysis of the LONP1 protein with this compound heterozygous mutation is recommended and may result in additional information about the pathogenetic mechanism of CODAS syndrome.

**TABLE 1:** The summary of reported *LONP1* variants in patients with CODAS syndrome.

Variation	p.A670V; p.R672C	p.R15P	p.R672C	p.S100Qfs*46; p.R786W	p.P749S; p.G767E	p.E476A; p.P749S	p.A670V; p.I927del
Zygosity	CHet	Hom	Hom	CHet	CHet	CHet	CHet
Number of cases (%)	1	2	5	1	1	2	1
References	This study	Khan et al. (29408517)	Khan et al. (29408517)	Inui et al. (28148925)	Dikoglu et al. (25808063)	Dikoglu et al. (25808063)	Dikoglu et al. (25808063)
		Patel et al. (27878435)				Yoo SD et al. (31169704)	
Cerebral							
Intellectual disability	1 (100%)	-	-	1 (100%)	1 (100%)	2 (100%)	-
Hypotonia and motor delay	1 (100%)	-	-	1 (100%)	1 (100%)	2 (100%)	-
Developmental delay	-	2 (100%)	-	1 (100%)	1 (100%)	2 (100%)	-
Spasticity	-	-	-	1 (100%)	-	-	-
Epilepsy	-	-	-	1 (100%)	-	-	-
Ocular							
Cataracts	1 (100&)	2 (100%)	2 (40%)	1 (100%)	1 (100%)	2 (100%)	1 (100%)
Pseudophakia	-	-	-	-	-	-	-
Ptosis	-	-	3 (60%)	-	-	-	-
Dental							
Caries	1 (100%)	-	-	-	-	-	-
Enamel dysplasia	-	-	-	-	-	-	-
Delayed tooth eruption	-	-	-	-	-	-	-
Dens hypoplasia	-	-	-	-	-	-	-
Auricular							
Scapha and helix dysplasia (‘‘Crumpled’’ ears)	1 (100%)	-	2 (40%)	-	1 (100%)	1 (50%)	-
Prominent columella	-	1 (50%)	3 (60%)	-	-	-	-
Elongated ear lobules	-	-	1 (20%)	-	-	-	-
Conductive hearing loss	-	-	-	-	-	-	-
Sensorineural hearing loss	-	-	-	-	-	-	-
Skeletal							
Genu valgus	1 (100%)	1 (50%)	3 (60%)	-	1 (100%)	1 (50%)	1 (100%)
Short stature	-	2 (100%)	-	1 (100%)	-	-	-
Scoliosis	-	-	-	-	-	-	-
Pes valgus	-	-	-	-	-	-	-
Vertebral coronal clefts	-	-	-	-	-	-	-
Skeletal dysplasia	1 (100%)	1 (50%)	-	-	1 (100%)	2 (100%)	1 (100%)

Variation	p.R672C	p.A670V	p.W464*; p.R679H	p.R721G	p.P676S
Zygosity	Hom	Hom	CHet	Hom	Hom
Number of cases (%)	1	1	1	4	1
References	Dikoglu et al. (25808063)	Dikoglu et al. (25808063)	Dikoglu et al. (25808063)	Strauss et al. (25574826)	Shebib et al. (1887855)
					Strauss et al. (25574826)
Cerebral					
Intellectual disability	-	-	1 (100%)	2 (50%)	1 (100%)
Hypotonia and motor delay	-	1 (100%)	1 (100%)	4 (100%)	-
Developmental delay	-	-	1 (100%)	-	1 (100%)
Spasticity	-	-	-	-	-
Epilepsy	-	-	-	1 (25%)	-
Ocular					
Cataracts	1 (100%)	1 (100%)	1 (100%)	4 (100%)	1 (100%)
Pseudophakia	-	-	-	-	-
Ptosis	-	-	-	4 (100%)	1 (100%)
Dental					
Caries	-	-	-	-	-
Enamel dysplasia	-	-	-	2 (50%)	-
Delayed tooth eruption	-	-	1 (100%)	2 (50%)	1 (100%)
Dens hypoplasia	-	-	1 (100%)	2 (50%)	1 (100%)
Auricular					
Scapha and helix dysplasia (‘‘Crumpled’’ Ears)	-	1 (100%)	1 (100%)	4 (100%)	1 (100%)
Prominent columella	-	-	-	-	-
Elongated ear lobules	-	-	-	-	-
Conductive hearing loss	-	-	-	3 (75%)	-
Sensorineural hearing loss	-	-	-	2 (50%)	1 (100%)
Skeletal					
Genu valgus	1 (100%)	-	1 (100%)	4 (100%)	-
Short stature	-	1 (100%)	1 (100%)	4 (100%)	1 (100%)
Scoliosis	-	-	-	4 (100%)	-
Pes valgus	-	-	-	2 (50%)	-
Vertebral coronal clefts	-	1 (100%)	1 (100%)	2 (50%)	-
Skeletal dysplasia	1 (100%)	1 (100%)	1 (100%)	4 (100%)	1 (100%)

CHet, compound heterozygous; Hom, homozygous.

## Conclusion

We used WES to explore the genetic entity in a Chinese boy who has suffered from cognition impairment, cataracts, caries, abnormal auricle and skeletal anomalies since birth. The patient’s parents are non-consanguineous and healthy. A compound heterozygous missense mutation (NM_004793: c.2009C>T/p.A670V and c.2014C>T/p.R672C) of *LONP1* was identified in the patient. The patient was diagnosed as CODAS syndrome. Here we reported the first case with CODAS syndrome in Chinese population. Our study not only provided data for genetic counseling and prenatal diagnosis to this family, but also supplied the typical clinical pictures of CODAS syndrome, which may contribute to the understanding and diagnosis of the disease combined with genetic analysis.

## Data Availability

The data presented in the study are deposited in the GSEHuman repository, accession number HRA002969 (https://ngdc.cncb.ac.cn/gsa-human/browse/HRA002969).
